# Relationships between Anxiety, Attention, and Reading Comprehension in Children

**DOI:** 10.21203/rs.3.rs-3088436/v1

**Published:** 2023-07-03

**Authors:** Emily D. Barnes, Amie E. Grills, Sharon R. Vaughn

**Affiliations:** Boston University; Boston University; University of Texas at Austin

**Keywords:** Anxiety, Attention, Reading, Comprehension, Executive Function

## Abstract

Many studies link anxiety in children with reading difficulties, but some facets of anxiety have been found to be positively associated with reading achievement. Attentional Control Theory offers a potential explanation for these seemingly contradictory findings, positing that anxiety can both interfere in attentional processes and enhance effort and use of compensatory processing strategies. The current study examines the relationships between anxiety, attentional control, and reading comprehension in a racially-diverse sample of 251 second-grade students, most of whom were struggling readers. Results showed that harm avoidance was positively associated with reading comprehension and physical symptoms of anxiety were negatively associated with reading comprehension. These links were attenuated when including attentional control in the model, suggesting mediation and lending support to Attentional Control Theory. Further research is needed to confirm causal mediation effects between anxiety, attentional control, and reading performance.

## Introduction

Anxiety is one of the most common and early onset mental health problems in youth (Grills-Taquechel & Ollendick, 2012; Merikangas et al., 2010b). Approximately one third of US adolescents have had an anxiety disorder in their lifetimes, and the median age of onset among these adolescents is 6 years old, coinciding with the beginning of elementary school (Merikangas et al., 2010b). However, less than one third of US youth with an anxiety disorder receives any mental health treatment, and younger children are less likely than adolescents to receive services (Merikangas et al., 2010a). Anxiety is a pervasive mental health problem for children, and it may also interfere with educational outcomes (Grills-Taquechel et al., 2012).

Consistent findings report a negative association between anxiety and reading achievement in children (Francis et al., 2019; Grills-Taquechel et al, 2012). Reading is an important educational target in elementary school and a predictor of academic achievement longer-term (Suggate et al., 2018). As such, anxiety represents a promising area of research for understanding how best to support reading achievement in early education (Grills-Taquechel et al., 2012). Prior research suggests that associations between anxiety and reading are bi-directional, with reading abilities influencing later anxiety levels and anxiety levels influencing later reading abilities (Grills-Taquechel et al., 2012; Ramirez et al., 2019).

Reading performance in children may also be associated with particular subtypes of anxiety (Grills-Taquechel et al., 2012). Poorer reading outcomes have been associated with elevations in separation anxiety and generalized anxiety disorder symptoms, but not specific phobia symptoms (Carroll et al., 2005). A study by Grills-Taquechel et al. (2012) found that reading fluency at the beginning of the school year negatively predicted separation anxiety symptoms at the end of the school year in first grade students. However, the same study found that harm avoidance, a facet of anxiety related to perfectionism, was *positively* associated with reading achievement, illustrating that certain facets of anxiety may play a motivating or performance-enhancing role (Grills-Taquechel et al., 2012).

Most studies examining the links between anxiety and reading performance have focused on reading skills such as decoding and fluency, with few examining the more complex skill of reading comprehension (Macdonald et al., 2021). Successful passage comprehension requires several cognitive tasks, including decoding words, reading fluently (with speed and accuracy), understanding word meanings (vocabulary), linking the text with prior knowledge, and monitoring one’s understanding of the text (Edmonds et al., 2009). One study of fourth and fifth grade struggling readers found that anxiety was more strongly associated with poorer reading comprehension than with basic reading skills (Macdonald et al., 2021). Additionally, this study found that domain-specific reading anxiety uniquely predicted reading comprehension, whereas generalized anxiety did not predict reading outcomes (Macdonald et al., 2021).

## Attentional Control Theory

Many of the findings connecting anxiety and reading are consistent with Attentional Control Theory (ACT; Eysenck et al., 2007), which posits that anxiety consumes cognitive resources, reducing performance on certain tasks. According to the theory, anxiety reduces goal-directed, top-down attentional control in favor of the bottom-up, stimulus driven system of attention (Eysenck & Derakshan, 2011). For example, a child trying to read may become distracted by a worried thought or anxiety-related stomachache, making it difficult to concentrate on reading. A recent meta-analysis of fifty-eight studies of children and adults largely supported the hypotheses of ACT (Shi et al., 2019). The effect size of anxiety on attentional control appears to be larger for tasks requiring higher cognitive loads compared to lower (Shi et al., 2019). As Macdonald et al. (2021) note, this effect may explain why anxiety reduces performance on comprehension to a greater extent than decoding and fluency, as comprehension is a more complex task requiring more cognitive resources.

Research examining the link between anxiety and attentional control often does not differentiate state and trait anxiety, though researchers generally use trait anxiety questionnaires as measures (Myles et al., 2020). Since individuals high in trait anxiety are more likely to experience state anxiety during a given task, it is unclear if findings linking trait anxiety with impaired attentional control reflect a stable difference in cognition in individuals high in trait anxiety or a temporary impairment in functioning caused by high state anxiety (Myles et al., 2020). One study of adults found that induced state anxiety was associated with impaired attentional control in individuals high in trait anxiety, but not in those low in trait anxiety (Myles et al., 2020). Thus, state and trait anxiety may interact to influence attentional control in adults. In children, there is a lack of research differentiating the impact of state and trait anxiety on attentional control. However, one study found that domain-specific reading anxiety was more strongly associated with poorer reading achievement than other anxiety subtypes, suggesting that state anxiety likely plays a key role, interfering in the moment with cognitive processes during reading (Macdonald et al., 2021).

In addition to explaining negative links between anxiety and cognition, ACT also states that anxiety may enhance effort and facilitate the use of compensatory processing strategies, which can protect against its deleterious effects on cognitive performance in some situations (Eysenck et al., 2007). Eysenck et al. posited that for this reason, anxiety may impair efficiency more than accuracy, so that a highly anxious person may put forth greater effort yet achieve the same results.

## Anxiety, Reading, and Attentional Control Theory in Children

Though many studies support ACT, few have tested its hypotheses as they may apply to anxiety and reading performance in children (Shi et al., 2019). Blankenship et al. (2019) found that executive function, closely related to attentional control, was directly associated with reading performance in 6-year-old children. However, this study did not include a measure of anxiety. Carroll et al. (2005) found that children with separation anxiety disorder or generalized anxiety disorder were more likely to have reading problems than their peers without anxiety disorders. Attention partially accounted for this anxiety-reading association, supporting the possibility that attentional control at least partially mediates the relationship between anxiety and reading performance.

A few studies directly examined anxiety and attention in the context of academic performance. Two studies have used dot-probe tasks to test attentional bias toward academic stressors in youth. Scrimin et al. (2016) found that eighth grade students who rated their school anxiety as higher showed greater bias toward academic stressors, supporting the hypothesis of ACT that individuals with anxiety have a harder time disengaging from threatening stimuli (Scrimin et al., 2016). However, this study did not examine associations between reading and anxiety. Another study of children ages 9 to 16 years found that those with specific learning disorders (mostly reading disorders) had a significant threat bias *away* from reading-related stimuli compared to typically-developing peers (Haft et al., 2019). While ACT would predict an attention bias *toward* threat, the results in this study are likely due to methodological choices (Haft et al., 2019; MacLeod et al., 2019). Specifically, a long presentation time in the dot-probe paradigm is more likely to reveal top-down attention rather than tapping into the bottom-up, stimulus-driven attentional processes that concern ACT (MacLeod et al., 2019). The longer presentation time was chosen for this study since children had low word-reading ability; however, the choice makes this study inconclusive regarding the hypotheses of ACT (Haft et al., 2019). In a study of first grade children, teachers rated children’s attention levels and children completed anxiety and reading measures (Grills-Taquechel et al., 2013). Among children rated as more attentive mid-year, elevated mid-year separation anxiety was associated with poorer end-of-year reading, whereas elevated mid-year harm avoidance was associated with better end-of-year reading (Grills-Taquechel et al., 2013). It is possible that these findings reflect the cognitive interference caused by separation anxiety in children who usually show good attentional control, or enhanced effort put forth by children high in harm avoidance.

## Present Study

Currently, there is evidence suggesting that attentional control could explain the links between anxiety and reading achievement; however, there is a lack of research testing this hypothesis. Since ACT accounts for both impaired efficiency in some cognitive tasks and increased effort and use of auxiliary strategies, it may be that certain facets of anxiety, such as harm avoidance, are particularly related to performance-enhancing aspects of anxiety, while other facets of anxiety are more related to cognitive interference. Though there is support for ACT in children, the theory has not been tested to explain the positive and negative relationships between subtypes of anxiety and reading in children. This knowledge could help us to understand facets of anxiety that are adaptive and maladaptive for attention and, in turn, reading, which has implications for designing interventions that can best support both mental health and academic achievement for students.

The current study presents an analysis of data collected during a reading intervention study with a diverse sample of public elementary school students in the Southwestern US (see Denton et al., 2014 for details). The study aims to test the hypotheses that different subtypes of anxiety will be differentially associated with children’s reading comprehension performance (H1), and the links between anxiety symptoms and reading comprehension will be attenuated when accounting for attention (H2). Specifically, for H1 we hypothesize that harm avoidance will be positively associated with reading comprehension and other subtypes of anxiety (social anxiety, separation anxiety, and physical symptoms) will be negatively associated with reading comprehension in elementary school students. Regarding H2, we predict that harm avoidance will be positively associated with attention, and that when attention is included in the model, the positive relationship between harm avoidance and reading comprehension will be attenuated. For all other anxiety subscales, we predict they will be negatively associated with attention, and when attention is included in the model, the negative relationship between these subscales and reading comprehension will be attenuated. This study evaluates whether attentional control serves as a potential mediator between anxiety and reading performance.

Additionally, the current study examines whether gender moderates the associations between anxiety, attention, and reading comprehension. A wealth of literature documents gender differences in attention and anxiety in children. In a large community sample, boys were rated as significantly more inattentive than girls by their parents, and boys are more than twice as likely as girls to be diagnosed with ADHD (Ramtekkar et al., 2010; Merikangas et al., 2010). In contrast, girls report higher levels of anxiety than boys, and this difference persists through adulthood (de Lijster et al., 2017). In contrast, reading performance in second-grade children appears unrelated to gender (Vlachos & Papadimitriou, 2015). Thus, it is important to examine whether gender moderates the relationships between anxiety, attention, and reading performance in this study.

## Methods

### Participants

Participants for this study were drawn from a larger randomized controlled trial examining reading interventions for children (see Denton et al., 2014, for details). Students were recruited from two school districts in the Southwestern US. Five urban schools participated from one district and four suburban or rural schools participated from the other district. In order to recruit an adequately powered sample for the larger study, two cohorts of students were recruited. All first grade students at participating schools were screened for reading difficulties at the end of the school years (*n* = 1,942). A sample of 251 children (*n* = 124 female) who had advanced to second grade were included in this study.

Participants were classified as struggling readers (*n* = 152) or typical readers (*n* = 99), based on their performance on standardized reading measures. Struggling readers were students who scored below the 30^th^ percentile (i.e., standard score < 93) on either the Test of Word Reading Efficiency (TOWRE; Torgesen et al., 1999) or the Woodcock-Johnson III Basic Reading Skills Composite (WJBR; Woodcock et al., 2001); typical readers scored above the 30^th^ percentile on both measures. This threshold has been validated against external criteria as a measure of response to reading intervention at the end of first grade (Fletcher et al., 2014).

The mean age of participants was 7.95 years (*sd* = .47) when they completed internalizing symptom measures and reading measures at the beginning of the 2^nd^ grade school year. Across participants, 49.4% identified as Black or African American, 30.3% Latinx or Hispanic, 6.4% non-Hispanic White, 0.8% Asian, and for 13.1% of participants, race and ethnicity was unknown. Among students for whom special education status was known (*n* = 171), 7% were receiving special education services.

### Measures

#### Gates-MacGinitie Reading Test Passage Comprehension

The Gates-MacGinitie Reading Test Passage Comprehension subscale (GMRT PC; MacGinitie et al., 2000) is a standardized assessment of passage comprehension ability, normed against national samples of children. The GMRT is a widely used, valid, and reliable instrument with alternate form reliability ranging from .80 to .87 (MacGinitie et al., 2000). Nationally-normed percentile rank scores were used in this study.

#### SWAN Inattention

The Strengths and Weaknesses of Attention-Deficit/Hyperactivity-Symptoms and Normal-Behaviors (SWAN; Swanson et al., 2012) rating scale is an evaluation based on observations of attention skills in diverse samples. The SWAN has good reliability and validity (Brites et al., 2015). The SWAN is comprised of 18 items, 9 of which make up the Inattention subscale and 9 of which make up the Hyperactivity/Impulsivity subscale. The SWAN is completed by a parent or teacher and captures variance at the positive and negative ends of the symptom dimensions (Swanson et al., 2012). The rater is asked to compare the child to others of the same age and school environment on skills such as paying attention to detail, listening when spoken to, and remembering daily tasks. Each item is scored on a 7-point Likert scale from 1 (below average attention) to 7 (above average attention). Mean item scores for the teacher-rated Inattention subscale are presented in this study and conceptualized as on-task attention. Internal consistency for the Inattention subscale in the present study was high (*a* = 0.98).

#### Multidimensional Anxiety Scale for Children

The Multidimensional Anxiety Scale for Children (MASC; March et al., 1997) is a 39-item child self-report measure of anxiety. Each item is rated using a 4-point Likert scale, ranging from “Never true about me” (0) to “Often true about me” (3). The MASC has a four-factor structure, with subscales of Physical Symptoms, Harm Avoidance, Social Anxiety, and Separation/Panic (March et al., 1997). The MASC shows good to excellent internal consistency and test-retest reliability (March, et al., 1999). The MASC was originally normed on children ages 8 years and older, but the developer sanctioned using the scale with ages 6 years and older if items are read out loud and age 8 norms are used (J. March, personal communication, May 25, 2007). Several studies have used the scale with 6- to 7-year-old children, finding that internal consistency and goodness-of-fit of the four-factor model are similar in this age group and in older cohorts (Grills et al., 2014; Langer et al., 2010; March et al., 1999). In the present study, internal consistency was acceptable (*a* = 0.61–0.89). Mean item scores for each subscale are presented in this study.

### Procedures

Study procedures were reviewed and approved by the universities’ committees for the protection of human subjects. Children’s parents received a letter of informed consent explaining study information and procedures. Children were read an assent statement and given the choice to opt out. All children assented to participating. Assessments were administered around the middle and end of the school year during which students received the intervention. Study coordinators trained examiners to administer assessments according to a manual and observed to ensure procedures were followed. Assessments occurred in quiet rooms in students’ own schools. Items on the MASC were read out loud and children were asked to rate each item silently on their answer sheet, with time provided for questions. Children were told to only look at their own answers, were allowed desk shields to keep their responses private, and were monitored by study staff to ensure confidentiality. Around the same time as the student assessments, English Language Arts teachers were asked to complete the SWAN for children in their classes.

### Data Analyses

Cross-sectional data from the first time point was used for these analyses due to theoretical considerations. Specifically, ACT suggests that state anxiety causes cognitive interference and decreases attentional control in the moment when a person is anxious (Eysenck et al., 2007; Myles et al., 2020). Because this hypothesized effect of anxiety on attentional control would occur nearly simultaneously, the use of longitudinal data (e.g., examining anxiety at mid-semester and attention at end of semester) would not tap into the research question this study aims to examine. The first time point was used in this study to examine the baseline relationships between anxiety, attention, and reading prior to the intensive reading intervention.

Statistical analyses were conducted using R version 4.1.1. First, independent samples t-tests were run to test for gender differences for the anxiety subscales, passage comprehension, and attention subscale. Next, the hypothesis that passage comprehension would be positively related to harm avoidance and negatively related to physical symptoms, social anxiety, and separation anxiety was tested with bivariate correlations. Correlations were run separately for male and female students. As data tended to be missing for individual measures only (e.g., teacher did not complete attention measure, child absent during individual testing session), pairwise exclusions were used in these analyses.

The hypothesis that the links between anxiety subtypes and reading comprehension are attenuated when accounting for attention was tested with mediation regression analyses with version 4.1 of the PROCESS macro for R, using model 4 and 5,000 bootstrapping resamples (Hayes, 2022). The mediation model included all anxiety subscales as independent variables. Gender was included as a covariate in the mediation model. Participants with complete data across these variables were included in the analyses (*n* = 243). For the anxiety subscales for which attention was a possible mediator of the relationship between anxiety and reading comprehension, moderated mediation models were run using gender as a moderator to explore these relationships further. Model 59 of the PROCESS macro and 5,000 bootstrapping resamples were used for the moderated mediation analyses (Hayes, 2022).

## Results

### Gender Differences

Gender differences for the MASC anxiety subscales, passage comprehension, and attention subscale are reported in Table 1. Using a Bonferroni adjusted alpha value of .008 (.05/6), independent samples *t*-tests revealed that female students reported higher anxiety than male students across the MASC harm avoidance, physical symptoms, and separation anxiety subscales. Female students were also rated as more attentive than male students by their teachers. After correcting for multiple comparisons, there were no significant gender differences on the MASC social anxiety subscale or passage comprehension scores.

### Bivariate Correlations

Pearson’s product-moment correlations were calculated for the relationships between the MASC subscales, passage comprehension, and attention (see Table 2). Given the observed gender differences, correlations were calculated separately for male and female students. All MASC subscales were correlated with one another in both male and female students (*ps* < .01). Harm avoidance was positively correlated with passage comprehension in male (*r* = .21, *p* = .023) and female students (*r* = .30, *p* < .001). In male students only, harm avoidance was also positively correlated with attention (*r* = .23, *p* = .010). No other MASC subscales were correlated with passage comprehension or attention (*ps* > .05). Attention was positively correlated with passage comprehension in male (*r* = .58, *p* < .001) and female students (*r* = .57, *p* < .001).

### Mediation Model

To test the hypothesis that the relationships between anxiety subtypes and reading comprehension are attenuated when accounting for attention, a mediation model was run examining the four subtypes of anxiety as independent variables. The anxiety subtypes were regressed on passage comprehension, with attention as the proposed mediator. Since all anxiety subscales were included in the regression, the calculated direct and indirect effects for each subscale represent the values when controlling for the other anxiety subscales. This approach was chosen since the subscales were found to be correlated with one another. Gender was also included as a covariate in all analyses (see Table 3).

#### Harm Avoidance as Independent Variable

As Table 3 shows, after controlling for gender and the other MASC subscales, harm avoidance was significantly positively related to passage comprehension (total effects model; *β* = 0.30, *p* < .001). Attention was also positively related to passage comprehension (*β* = 0.54, *p* < .001). When controlling for attention along with gender and the other anxiety subscales, there was a reduced direct effect of harm avoidance on passage comprehension (*β* = 0.17, *p* = .006). A bias-corrected bootstrap 95% confidence interval for the indirect effect based on 5,000 bootstrap samples did not include zero (*B* = 5.19, *CI* = [1.68, 8.94], *β* = 0.14), indicating the relationship between harm avoidance and passage comprehension was partially accounted for by teacher-rated attention, supporting the second hypothesis.

#### Physical Symptoms as Independent Variable

After controlling for gender and the other MASC subscales, the physical symptoms subscale was significantly negatively related to passage comprehension (total effects model; *β* = −0.28, *p* < .001; see Table 3). This finding supported the first hypothesis. When controlling for attention along with gender and the other MASC subscales, there was a reduced direct effect of physical symptoms on passage comprehension (*β* = −0.17, *p* = .015). A bias-corrected bootstrap 95% confidence interval for the indirect effect based on 5,000 bootstrap samples did not include zero (*B* = −4.31, *CI* = [−8.38, −0.64], *β* = −0.10). These results indicate that teacher-rated attention also partially accounted for the relationship between physical symptoms of anxiety and passage comprehension.

#### Separation Anxiety and Social Anxiety Subscales as Independent Variables

As shown in Table 3, the separation anxiety and social anxiety subscales were not related to passage comprehension in either the total effects or direct effects models (*p*s > .05). There was no evidence for indirect effects of these anxiety subtypes on passage comprehension via teacher-rated attention (95% CI for SEP = [−5.80, 2.83]; 95% CI for SOC = [−2.57, 4.45]). Thus, the first and second hypotheses were not supported for social anxiety and separation anxiety.

### Moderated Mediation Models

**Figure 1:**
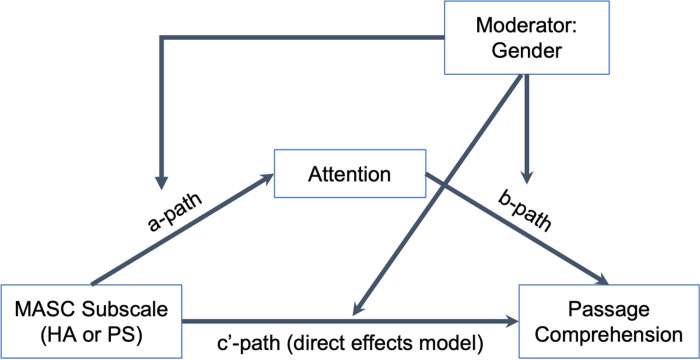
Moderated Mediation Model Tested Using PROCESS Model 59 *Note. HA* Harm Avoidance Subscale, *PS* Physical Symptoms Subscale.

Because gender was related to anxiety subscales (see Table 1), additional analyses were run examining gender as a moderator of the direct and indirect effects of the anxiety subscales on passage comprehension. Separate moderated mediation models were run with independent variables of harm avoidance and physical symptoms, the subscales for which there was evidence of an indirect effect of anxiety on passage comprehension via attention. These analyses examined whether gender moderated the relationships between the MASC subscale and attention (a-path), between attention and passage comprehension (b-path), and/or between the MASC subscale and passage comprehension (i.e., direct effects or c’-path). Figure 1 depicts the moderated mediation model that was tested for the harm avoidance and physical symptoms subscales separately.

In neither case was there a significant interaction between anxiety and gender on attention for the a-path or between attention and gender on passage comprehension for the b-path (see Table 4). The direct effect from harm avoidance to passage comprehension was also not moderated by gender and the index of moderated mediation was not significant in either analysis (see Table 4). Thus, there was no evidence of moderated mediation.

## Discussion

The primary aims of the present study were to: (1) test whether different subtypes of anxiety were differentially associated with children’s reading comprehension and (2) examine the potential mediating role of attention in these relationships. An additional exploratory aim was to examine whether gender moderated the relationships between anxiety, attention, and reading. Concurrent measures of anxiety, attention, and reading comprehension were used due to theoretical considerations; specifically, that anxiety likely impacts attention and cognitive performance on tasks in the moment it occurs (Myles et al., 2020; Macdonald et al., 2021). Results indicated that harm avoidance was positively related to passage comprehension, while physical symptoms were negatively related to passage comprehension. Both relationships were partially mediated by teacher-rated on-task attention. Gender did not moderate these mediations.

### Relationships between Anxiety Subtypes and Reading Performance

Overall, the first hypothesis that different facets of anxiety would be differentially related to reading comprehension was supported. Bivariate correlations revealed that harm avoidance was positively related to passage comprehension for both male and female students. This result is consistent with prior findings that harm avoidance was positively related to reading fluency and decoding in first grade students (Grills-Taquechel et al., 2012). A substantial literature indicates that anxiety can have a performance-enhancing role at moderate levels, including in academic performance (Yerkes & Dodson, 1908; Karaman et al., 2020). Perfectionism, which is closely related to harm avoidance, is sometimes negatively associated with mental health and performance, but sometimes positively associated with performance and emotion regulation strategies such as reappraisal (Smith et al., 2022; Vois & Damian, 2020). Children high in perfectionism may also engage in more metacognitive strategies when reading, which can aid comprehension (Bagri & Dickinson, 2023). Thus, consistent with ACT, harm avoidance may represent a motivating subtype of anxiety associated with improved cognitive performance.

Although there were no other significant bivariate correlations between anxiety subscales and reading comprehension in the present study, when all anxiety subscales were included in the regressions for the mediation model, the physical symptoms subscale was negatively associated with passage comprehension. Individuals with generalized anxiety disorder typically present with both physical symptoms of anxiety and worry, and past research is mixed on whether generalized anxiety symptoms are associated with poorer reading performance in children (Carroll et al., 2005; Macdonald et al., 2021). One possible explanation for these mixed findings is that most studies of children’s anxiety and reading performance have not separated physical symptoms of anxiety from worries. Indeed, a recent study found that children high in both physical symptoms of anxiety and worries performed worse on intelligence indices than children high in worries but low in physical symptoms (Castagna et al., 2021). Thus, it is critical to distinguish different dimensions of anxiety to understand how and when it is related to poorer cognitive or academic performance. The overall level of anxiety may be less related to reading performance than the specific subtypes of anxiety experienced. Further research should continue to examine interactions between different subtypes of anxiety.

The hypotheses that social and separation anxiety would be negatively related to reading performance were not supported in this study. Since social anxiety disorder typically emerges in early adolescence, additional research is needed to examine whether social anxiety interferes with reading performance in older students (de Lijster et al., 2017). Given the age of the students in the present study, it is likely that social anxiety was not a salient subtype of anxiety for most participants. Indeed, mean item scores were lowest for the social anxiety subscale of all the anxiety subscales (see Table 1). This finding is consistent with past research on social anxiety in young children (Grills et al., 2014).

Prior research on separation anxiety and reading achievement is mixed (Carroll et al., 2005; Grills et al., 2022; Grills-Taquechel et al. 2012). Grills et al. (2022) found that separation anxiety predicted non-response to reading intervention among struggling readers in second grade. However, the same study found that typical readers reported significantly higher separation anxiety at the beginning of the school year than struggling readers did. Thus, it may be that a moderate amount of separation anxiety is normative in this age group yet can interfere in learning for children who are already struggling readers. Additionally, Carroll et al. (2005) found that children with separation anxiety disorder were more likely to have specific literacy difficulties than children without separation anxiety disorder. Taken together, these studies suggest that children with elevated separation anxiety may be at a greater risk for reading problems, and for children with reading difficulties, elevated separation anxiety may be a risk factor for non-response to intervention (Carroll et al., 2005; Grills et al., 2022). However, when controlling for other facets of anxiety, such as physical symptoms, separation anxiety was not related to reading performance in the present study. Thus, additional research is needed to understand when separation anxiety is associated with poorer reading performance and how it may interact with other factors, such as physical symptoms and learning disabilities, to influence reading achievement.

Overall, the findings suggest that the behaviors measured by the harm avoidance subscale may represent a motivating dimension of anxiety, while physical symptoms may represent an interfering dimension, consistent with the hypotheses of ACT (Eysenck & Derakshan, 2011).

### Attention as a Mediator in the Anxiety-Reading Comprehension Relationships

The second aim of the study was to examine whether attention mediated the link between anxiety and reading comprehension. This work was grounded in ACT, which hypothesizes that anxiety impacts performance on cognitive tasks both by increasing effort and the use of auxiliary processing strategies, and by reducing attentional control and thus decreasing cognitive efficiency (Eysenck & Derakshan, 2011). The present study extended the body of literature on ACT by testing the specific hypotheses that (1) attention would be positively related to harm avoidance and mediate the relationship between harm avoidance and passage comprehension and (2) attention would be negatively related to physical symptoms and mediate the relationship between physical symptoms and passage comprehension. Both specific hypotheses were supported by the results of the mediation analysis. Attention partially mediated the positive relationship between harm avoidance and reading comprehension and the negative relationship between physical symptoms and reading comprehension.

These findings may help to make sense of seemingly contradictory findings in prior research that have linked anxiety with both better attention and reading achievement and poorer attention and reading achievement. For example, harm avoidance was positively associated with attention in first grade students, which was in turn related to better achievement (Grills-Taquechel et al., 2013). Other studies have also supported the finding that anxiety is sometimes related to better academic performance (Fernández-Castillo & Gutiérrez-Rojas, 2009; Karaman et al., 2020). However, a recent review found that individuals with reading difficulties were more likely to experience anxiety problems (Francis et al., 2019). ACT may explain the conflicting results with its claims that anxiety can be both motivating and interfering. Eysenck et al. (2007) suggest that the level of anxiety and the difficulty of the cognitive task may be most important in determining whether anxiety has detrimental or helpful effects.

The present study suggests that it is not only the quantitative level of anxiety that is important in predicting a person’s performance on a cognitively difficult task; the qualitative nature of the anxiety and the particular subtypes of anxiety experienced likely play an important role, as well. Specifically, harm avoidance may be more associated with increased effort, enhanced on-task attention, and improved cognitive performance, and physical symptoms may be more associated with reduced attentional control and cognitive performance. This understanding is critical for making sense of the contradictory findings regarding the relationship between anxiety and reading performance in children. Additional research is needed to explore whether harm avoidance is associated with increased effort, which may in turn impact attentional control and reading performance.

### Role of Gender in Anxiety-Attention-Reading Relationships

Though there were gender differences in levels of anxiety with girls reporting higher anxiety in the present study, there was no evidence that gender moderated the mediation between anxiety, attention, and passage comprehension. A great deal of research corroborates the finding that girls report higher levels of anxiety than boys (de Lijster et al., 2017). Similarly, the finding that gender was not a moderator in this study was unsurprising, as gender differences are typically not reported in the literature on ACT (Shi et al., 2019). However, given that girls report higher anxiety and teachers rated girls as more attentive in this study, this null finding was important in establishing that the attention-enhancing and attention-reducing effects of different facets of anxiety likely occur for both boys and girls with elevated anxiety.

### Limitations, Future Directions, and Implications

The present study has several limitations. Establishing causal mediation requires several steps, not all of which this study could satisfy. While this study establishes correlations, it does not establish temporal precedence or the removal of confounding variables (Hayes, 2022). Cross-sectional data was used due to theoretical considerations and empirical evidence suggesting state anxiety impacts attentional control in the moment, leading to nearly instantaneous cognitive interference (Myles et al., 2020; Macdonald et al., 2021). This cross-sectional analysis also cannot account for all possible confounding variables, as is true for longitudinal observational research, as well. However, the independent and dependent variables and the covariates for the model in this study were selected using a theory-driven approach. Attention attenuated the relationships between anxiety subscales and reading comprehension in this study. As such, results indicate that attention is a possible mediator between anxiety and reading performance; further research is needed to establish a causal relationship.

The measures in this study also present limitations. The teacher-rated attention scale broadly measures children’s attentional functioning in school, but it does not provide information about the specific cognitive processes, such as inhibitory control or set-shifting, thought to be impacted by anxiety, according to ACT (Eysenck et al., 2007). Though questionnaires likely reflect children’s daily functioning accurately, their validity in measuring cognitive processes is unclear, as they do not correlate well with efficiency or effectiveness on behavioral tasks (Blijd-Hoogewys et al., 2014). Measures of attentional control completed by proxy reporters such as teachers may also be influenced by other variables, such as a child’s misbehavior (Álvarez-García et al., 2014). Additionally, teacher, parent, and child ratings of externalizing symptoms correspond only moderately (De Los Reyes et al., 2015). Thus, future research incorporating parent and child ratings of attention, along with behavioral measures of cognitive processes such as inhibitory control and set-shifting, would help to provide a fuller picture of the relationships between anxiety, attentional control processes, broad attentional functioning, and reading comprehension. Alongside robust attention measures, future studies would benefit from including a domain-specific reading anxiety measure and a multidimensional anxiety measure to clarify which facets of anxiety most strongly relate to reading performance.

Results of this study point to additional directions for future research. Given that attention only partially mediated the anxiety-reading links, it seems likely that problems in attention make up part but not all of the relationship between high anxiety and reading problems described in the literature. Researchers must consider what other factors may also contribute. For example, certain subtypes of anxiety may reduce reading self-efficacy, which may in turn reduce reading performance. Additional research is also needed to understand whether different dimensions of anxiety are associated with academic performance in youth of different ages and with different academic challenges (e.g., math difficulties). For example, social anxiety may be more cognitively interfering for adolescents than for young students. Potential moderating factors such as working memory and the use of meta-cognitive strategies should also be examined. Meta-cognitive strategies may moderate the positive relationships between harm avoidance and attention or reading comprehension. Additionally, more studies comparing general and clinical populations of children are needed to understand these relationships in children with anxiety disorders and specific learning disabilities.

Finally, the present study lays the groundwork for an experimental study directly testing ACT as an explanation for the links between anxiety and reading performance. Specifically, a randomized experimental study examining the relationships between anxiety, effort, attention, and reading comprehension before and after anxiety treatment would help to strengthen understanding of these relationships in an ecologically valid context. For example, reductions in certain dimensions of anxiety, such as physical symptoms, may be more meaningful in targeting cognitive interference. Further work in this area has important implications for designing reading interventions that address anxiety-related cognitive interference and improve children’s emotional well-being.

## Figures and Tables

**Table 1:** Means by Gender and Independent Sample t Tests

Variable	Female *M (SD)*	Male *M (SD)*	*t(df)*	*p*
MASC HA	2.07 (0.57)	1.85 (0.71)	2.72 (230)	.007*
MASC PS	1.40 (0.57)	1.16 (0.59)	3.22 (242)	.001*
MASC SOC	1.28 (0.7)	1.06 (0.68)	2.49 (243)	.014
MASC SEP	1.47 (0.57)	1.14 (0.56)	4.48 (243)	< .001*
PC	31.10 (25.06)	24.88 (23.01)	2.02 (241)	.044
Attention	4.31 (1.39)	3.53 (1.46)	4.27 (242)	< .001*

*Note. MASC* Multidimensional Anxiety Scale for Children, *HA* Harm Avoidance Subscale, *PS* Physical Symptoms Subscale, *SOC* Social Anxiety Subscale, *SEP* Separation Anxiety Subscale, *PC* Gates-MacGinitie Passage Comprehension. Bonferroni corrected

* *p* < .008.

**Table 2:** Pearson’s Correlations with Confidence Intervals by Gender

Variable	1	2	3	4	5	6
1. MASC HA	–	.33**	.29**	.46**	.30**	.16
		[.16, .48]	[.12, .44]	[.31, .59]	[.13, .45]	[−.01, .33]
2. MASC PS	.28**	–	.62**	.52**	−.13	−.14
	[.10, .43]		[.50, .72]	[.38, .64]	[−.30, .04]	[−.31, .03]
3. MASC SOC	.45**	.64**	–	.55**	−.02	.01
	[.29, .58]	[.52, .74]		[.42, .66]	[−.19, .16]	[−.17, .19]
4. MASC SEP	.40**	.55**	.68**	–	.03	.05
	[.24, .54]	[.41, .66]	[.57, .76]		[−.14, .21]	[−.13, .23]
5. PC	.21*	−.16	.03	−.01		.57**
	[.03, .37]	[−.33, .02]	[−.15, .21]	[−.19, .17]	–	[.44, .68]
6. Attention	.23*	−.09	−.03	−.09	.58**	
	[.06, .40]	[−.26, .09]	[−.21, .15]	[−.27, .09]	[.45, .69]	–

*Note. MASC* Multidimensional Anxiety Scale for Children, *HA* Harm Avoidance Subscale, *PS* Physical Symptoms Subscale, *SOC* Social Anxiety Subscale, *SEP* Separation Anxiety Subscale, *PC* Gates-MacGinitie Passage Comprehension. Values above the diagonal represent correlations for female students and values below the diagonal represent correlations for male students. Values in square brackets indicate the 95% confidence interval for each correlation.

* indicates *p* < .05.

** indicates *p* < .01.

**Table 3:** Mediation Model Testing for Anxiety Subscales, Attention, and Passage Comprehension

Predictor (IV)	*B*	SE	95% CI		*p*	
			Lower	Upper		*β*
*Total effects (IV → passage comprehension)*						
HA	11.40	2.57	6.33	16.46	<.001	0.30
PS	−11.46	3.39	−18.14	−4.78	<.001	−0.28
SEP	−0.45	3.54	−7.42	6.51	.898	−0.01
SOC	2.53	3.04	−3.46	8.52	.406	0.07
Gender	6.01	3.08	−0.06	12.08	.052	0.12
*Direct effects (IV → passage comprehension)*						
HA	6.21	2.24	1.80	10.62	.006	0.17
PS	−7.15	2.90	−12.86	−1.43	.015	−0.17
SEP	0.92	2.99	−4.97	6.82	.758	0.02
SOC	1.65	2.57	−3.42	6.71	.523	0.05
Attention	8.87	0.91	7.08	10.66	<.001	0.54
Gender	−1.16	2.71	−6.49	4.17	.669	−0.02
*Indirect effects (IV → attention → passage comprehension)*						
HA	5.19	1.85	1.68	8.94		0.14
PS	−4.31	2.00	−8.38	−0.64		−0.10
SEP	−1.38	2.19	−5.80	2.83		−0.03
SOC	0.89	1.79	−2.57	4.45		0.03

*Note. HA* Harm Avoidance Subscale, *PS* Physical Symptoms Subscale, *SEP* Separation Anxiety Subscale, *SOC* Social Anxiety Subscale. *B* represents unstandardized regression coefficient, *β* represents standardized regression coefficient. For total effects and direct effects, ordinary least squares standard errors (SE) and confidence intervals (CI) are shown. For indirect effect, bootstrap SE and bias-corrected bootstrap CIs are shown.

**Table 4:** Regression Results for the Moderated Mediation Analyses

	*Moderated Mediation Analysis 1: Harm Avoidance Subscale as the IV*					
	DV: Attention			DV: Passage Comprehension		
Predictor	*B*	SE	*p*	*B*	SE	*p*
HA	0.48	0.18	.009	2.42	2.57	.348
Gender	0.88	0.61	.150	−19.83	10.09	.051
HA × Gender	−0.10	0.29	.742	7.25	4.10	.079
Attention				8.96	1.25	<.001
Attention × Gender				0.75	1.79	.675
	*Moderated Mediation Analysis 2: Physical Symptoms Subscale as the IV*					
	DV: Attention			DV: Passage Comprehension		
Predictor	*B*	SE	*p*	*B*	SE	*p*
PS	−0.22	0.22	.324	−4.32	3.08	.162
Gender	1.11	0.45	.015	−7.96	10.07	.430
PS × Gender	−0.18	0.32	.565	2.36	4.47	.597
Attention				9.08	1.24	<.001
Attention × Gender				1.09	1.80	.543

*Note. HA* Harm Avoidance Subscale. *PS* Physical Symptoms Subscale. *B* represents unstandardized regression coefficient. Ordinary least squares standard errors (SE) are shown.

## Data Availability

Data is available on request.
